# Anticoagulating New-Onset Atrial Fibrillation After COVID-19: A Single-Center Experience

**DOI:** 10.7759/cureus.53909

**Published:** 2024-02-09

**Authors:** Miles Babb, Kurt Stevenson

**Affiliations:** 1 Internal Medicine, Boise Internal Medicine Residency, University of Washington, Boise, USA; 2 Infectious Diseases, Boise Veterans Affairs Medical Center, Boise, USA; 3 Internal Medicine, Division of Allergy and Infectious Diseases, University of Washington, Seattle, USA

**Keywords:** secondary atrial fibrillation, direct oral anticoagulant (doac), anticoagulation, inpatient, new-onset atrial fibrillation, covid-19

## Abstract

Anticoagulation (AC) strategy in new-onset atrial fibrillation (NOAF) secondary to other illnesses has not been broadly studied, and society-level guidance does not provide a strong recommendation regarding outpatient continuation upon discharge. Our study focused specifically on patients experiencing NOAF secondary to COVID-19. It sought to understand whether our facility’s rounding prescribers were continuing patients on AC at discharge, the presence of arrhythmia at one-year follow-up, and to observe the risk of adverse outcomes in light of this unique precipitant.

A retrospective cohort analysis and chart review were conducted of 231 consecutive inpatients during the initial 19 months of the COVID-19 pandemic. Eighteen patients experiencing NOAF with an average calculated CHA_2_DS_2_-VASc score of four were included in the cohort. Four patients (22%) died during hospitalization and 14 patients were discharged. Twelve of fourteen patients (86%) were discharged on AC, and eight remained adherent at follow-up. Two discharged patients died of unknown causes prior to follow-up. At follow-up, which occurred at a median of 1.2 years, 25% of the surviving cohort remained in atrial fibrillation (AF). No major bleeding events were recorded during the studied period.

This retrospective analysis of a small sample of patients admitted to a single medical center for COVID-19 and experiencing NOAF demonstrates that local prescribers are continuing AC at discharge, that the rate of recurrence of AF is similar to onset in non-COVID illness at one year, and that risk of death approximated that of COVID-19 itself rather than NOAF.

## Introduction

Outpatient anticoagulation (AC) strategy following secondary atrial fibrillation (AF) - that with a clearly defined precipitant - is not broadly studied, and society-level guidance provides only recommendations for discussion of its risks and benefits balanced against the risk of recurrence [1]. Coronavirus disease 2019 (COVID-19) is a novel pathogen that drives the onset of arrhythmia through various mechanisms including hypoxemia [2] and may result in a hypercoagulable state, for which (at the time of this study) patients often received therapeutic AC while admitted [3]. We sought to understand who among those admitted for COVID-19 and experiencing new-onset atrial fibrillation (NOAF) were being chronically anticoagulated upon hospital discharge and the risks of durable AC and arrhythmia presence at one year to the local population by analyzing local practice and outcomes.

Between 4-15% of patients hospitalized with COVID-19 may develop NOAF [4-6], which not only portends worsened in-hospital prognosis [7] but is an independent predictor of major adverse cardiac events and death [8,9]. While there exists information about best practices for patients already on AC prior to hospitalization for COVID-19 [10], expert guidance regarding *de novo* AC strategy upon hospital discharge serves only to support a discussion of risks and benefits balanced with the risk of AF recurrence [1].

We sought to (1) understand if local prescribers were placing patients on durable AC following the development of NOAF in COVID-19 illness, (2) observe the rate of resolution or ongoing presence of arrhythmia in the local population after onset during admission for COVID-19 and determine consistency with other non-COVID illness, and (3) observe the risk of a major bleeding event (MBE) or death in the target population at one-year follow-up.

## Materials and methods

A retrospective cohort analysis was performed via manual chart review with inclusion criteria by International Classification of Diseases (ICD) 10 code: patients admitted to Boise Veterans Affairs Medical Center for COVID-19 disease (U07.1) or other respiratory failures (J96.x) with positive COVID-19 polymerase chain reaction (PCR), diagnosis of atrial arrhythmia (I48.x), and a discharge date between March 1, 2020, and September 20, 2021, to allow sufficient lead time for one-year follow-up. Records meeting these criteria were furnished by the Veterans Affairs Informatics and Computing Infrastructure database.

**Figure 1:**
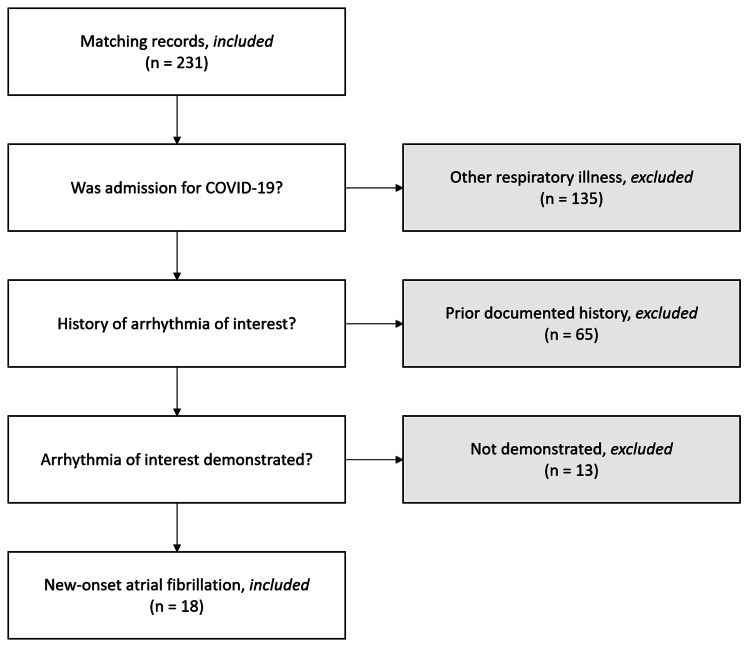
Chart exclusion flow diagram

We excluded those patients who had negative COVID-19 PCR testing, carried prior evidence of AF at the time of admission, and did not demonstrate evidence of AF during the hospital stay. Predetermined absolute exclusion criteria were age under 18 and current pregnancy at the time of admission, though no patient records matching these criteria were returned. Figure 1 is a flow diagram illustrating our chart exclusion process.

Once the cohort was identified, we underwent a manual chart review of the selected patients’ daily progress notes and discharge summaries, vital signs, and electrocardiogram data to identify those who had experienced NOAF during their admission. Selected laboratory studies pertinent to cardiac congestion, injury, and systemic inflammation at the time of arrhythmia onset (plus or minus one day) were also recorded. For cardiac structural data, the nearest available echocardiogram to the admission in question was utilized.

The follow-up interval was defined by the first visit available for review nearest the one-year date following discharge to determine the continuance of AC, presence or resolution of AF, and incidence of MBE or death. Visits earlier or later than one year were often utilized given sporadic healthcare contacts among the cohort.

Statistical analyses were single-tailed t-tests performed in Microsoft Excel, version 2302 (Microsoft Corporation, Redmond, United States). The review was deemed minimal risk and approved to proceed by both Puget Sound Veterans Affairs Internal Review Board and Boise’s local Research Development Committee (1714856).

## Results

Records of 231 consecutive inpatients were returned according to inclusion criteria. Eighteen remained in the review cohort after the application of exclusion criteria (Figure 1), the demographics of which are summarized in Table 1.

**Table 1:** Cohort demographics and biometric data ‡: De-identified for advanced age; †: Value obtained from technetium scan; *: Expired prior to follow-up HOD: Hospital day of onset; AC: Anticoagulation; LPM: Liters per minute; TTE: Transthoracic echocardiogram; LA: Left atrial; LAVI: Left atrial volume, indexed; LVEF: Left ventricular ejection fraction; BNP: B‐type natriuretic peptide

Patient	Age	Sex	HOD	CHA_2_DS_2_-VASc score	Oxygen requirement (LPM)	TTE time after arrhythmia (days)	LAVI (mL/m^2^)	LVEF (%)	BNP (pg/mL)	Troponin-T (ng/mL)	Follow-up interval (days)
1	74	Male	0	4	-	80	23.9	55-60	76	27	415
2	67	Female	0	3	-	14	42.2	65-70	110	8	485
3	75	Male	0	3	4	101	24.8	60-65	-	61	436
4	72	Male	1	4	-	288	42.0	65-70	104	14	317
5	>89^‡^	Male	4	3	6	-	-	-	-	-	*
6	87	Male	0	4	-	-	-	-	-	49	437
7	74	Male	0	1	-	37	24.6	60-65	95	6	631
8	79	Male	0	5	-	48	30.6	65-70	314	84	344
9	69	Male	0	3	-	33	44.3	60-65	296	13	97
10	81	Male	3	4	2	-	-	-	24	26	*
11	81	Male	0	4	30	47	26.9	50-55	875	111	649
12	>89^‡^	Male	6	5	3	-412	-	60-65	-	187	*
13	65	Male	0	2	-	1	-	60-65	273	27	*
14	76	Male	8	6	40	1	42.9	45-50	621	-	*
15	76	Male	0	4	5	64	-	55-60	136	27	509
16	>89^‡^	Male	0	3	-	-	-	-	85	17	*
17	73	Male	0	2	-	94	-	65-70	42	5	146
18	68	Male	0	5	-	-	-	70†	-	85	415

**Figure 2:**
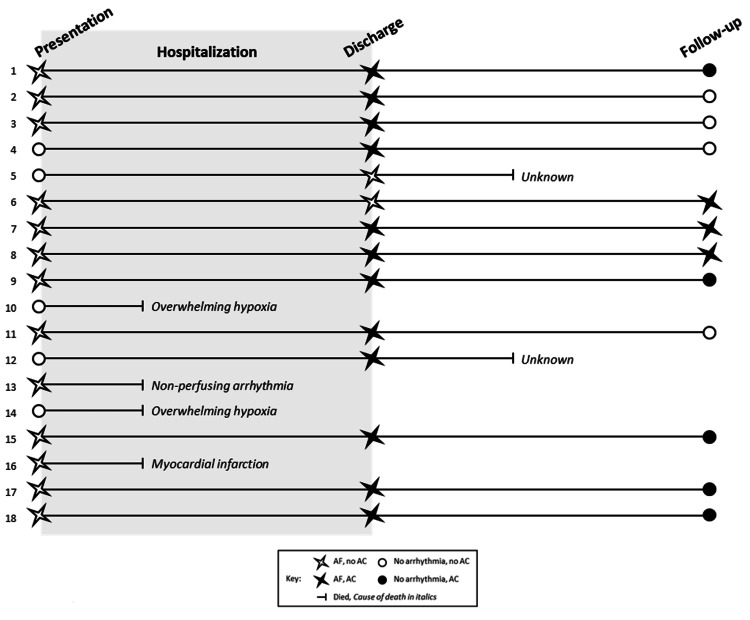
Individual course by patient

A graphical representation of each patient's individual course resides in Figure 2. Assigned numbers coincide with Table 1 above.

Nearly the entire cohort (17/18, 94%) were elderly males. Thirteen of eighteen (72%) patients presented with NOAF and the remainder experienced arrhythmia onset median of hospital day four. At arrhythmia onset 11/18 (61%) patients did not have a supplemental oxygen requirement. The average calculated CHA₂DS₂-VASc score was four, with a range of one to six.

Four patients (22%) died prior to discharge. About 13 patients had a CHA₂DS₂-VASc ≥2, only 11 of whom were discharged on AC. The sole patient with a CHA₂DS₂-VASc of one was also discharged on AC. All patients were initiated on direct oral anticoagulant. Two patients died after discharge but prior to follow-up, which occurred for the surviving cohort at a median of 1.2 years.

Aggregated median figures and statistical analyses comparing the resolution or persistence of AF groups are presented in Table 2.

**Table 2:** Comparison of groups at follow-up ¥: Calculated mean rather than median; *: Insufficient data to calculate

	Arrhythmia persistence	Arrhythmia resolution		
n	3	9		
Demographics at arrhythmia onset			Range (minimum, maximum)	p-value
Male (%)	100%	89%		
Age (years)	79	73	(65, 95)	0.03
CHA2DS2-VASc (points)^¥^	4	4	(1, 6)	
Hospital day of onset (days since presentation)	0	0	(0, 9)	
Laboratory values				
Troponin-T (ng/mL)	49	27	(5, 187)	0.39
D-Dimer (ng/mL)	0.62	1.16	(0.54, 4.05)	0.45
Procalcitonin (ng/mL)	0.06	1.82	(0.06, 3.57)	0.36
NT-proBNP (pg/mL)	205	110	(24, 875)	0.45
Fibrinogen (mg/dL)	391	1017	(391, 1148)	*
C-Reactive protein (mg/dL)	3.6	17.1	(0, 30)	0.07
Oxygen requirement (liters per minute)	0	0	(0, 40)	
Echocardiographic values				
Left atrial indexed volume (mL/m^2^)	27.6	26.9	(23.9, 44.3)	0.21
Left ventricular ejection fraction (%)	65	65	(45, 70)	*
Follow-up interval (days)	437	415	(97, 649)	0.47

At the time of follow-up, 3/12 (25%) patients remained in AF, and patients with resolution were significantly younger. Eight of twelve patients (75%), including all those with ongoing arrhythmia, remained on AC at follow-up. Notably, one patient (#11) did experience an embolic stroke after the studied period despite demonstrating no arrhythmia at follow-up. There were no MBEs recorded in any phase of care among the cohort.

## Discussion

This retrospective review gave beneficial insight into practice and prescribing patterns among local academic faculty and trainees and illuminated the risk of the ongoing presence of arrhythmia after admission for COVID-19 illness backed up by larger datasets.

A calculated median age of 75 indicates that our cohort was somewhat older than the national median for veterans and that the Vietnam War era was best represented, though our cohort ranged well into the World War II era. Given the representative demographics of servicemembers from these eras, it was no surprise that most of our cohort were male [11].

Despite society guidelines providing only 2b recommendations for the practice [1], 86% of patients in our cohort who may have benefited (by CHA_2_DS_2_-VASc score) were discharged on AC. It seems fair to conclude that this was reflective of the local prescribing culture during the studied 19-month period, which included most of our residents and rounding faculty during these initial COVID-19 surges.

The observed risk of ongoing AF (recurrence or persistence) in our cohort at one year was 25% among those surviving to follow-up, slightly elevated compared to Wang et al., who quantify the risk of recurrence after any precipitant as 20% at one year [12]. While we have focused solely on one type of respiratory illness, the presence of AF at one-year onset in COVID-19 specifically appears to approximate that in other acute infections (e.g., sepsis, influenza) despite our diminutive sample.

A modestly increased risk of stroke and mortality in secondary AF has been quantified in larger datasets [13]. That 28% of our small cohort expired prior to follow-up highlights the severity of COVID-19, especially considering their advanced age, and unfortunately seems to correlate with Centers for Disease Control data for COVID-19 admissions and deaths in the same period [14].

Limitations

Our study is limited by a small sample size, which makes any statistical conclusions prone to significant error. Echocardiographic data especially was limited both by availability and recency. Available data ranged from over a year prior to arrhythmia onset to more than nine months thereafter. Notably, five patients had no sonographic data available at all, which severely limits generalizability in a cohort of this size. Ideally indexed atrial volume at the time of arrhythmia onset would be compared between those with persistence versus resolution at follow-up given its association with AF [15], but sufficient echocardiographic data to examine this association was unavailable. Larger studies would be better suited to detect a significant structural difference between those with arrhythmia persistence versus resolution.

## Conclusions

Despite its small size, analysis of our retrospective cohort of patients experiencing NOAF during admission for COVID-19 illness demonstrates that local prescribers are placing patients on AC at discharge, that the rate of recurrence of AF at one year is similar to that of other non-COVID illnesses, and that incidence of poor outcome in this group was starkly elevated compared to all precipitants of secondary AF, but similar to that for COVID-19 illness nationwide during the study period.
